# A New Volume

**Published:** 1854-06

**Authors:** 


*A New Volume.*—Our present number inaugurates a new volume. It is unnecessary to repeat the usual assurances of distinguished regard for our readers, and of the permanence of our Journal. We intend to show the one by our efforts to furnish a readable and valuable monthly; the other is made manifest by an increasing subscription list. Our publishers, however, though very good-natured and long-suffering men, intimate that there is a large amount due them on subscriptions, some of them running back for a very disreputable number of years. We do not often allude to the business matters of the Journal, and only do so now, in the confidence that this notice will be sufficient to bring forward a goodly number of remittances, directed to Jewett, Thomas & Co., to whom all business letters should be addressed.

The editorial management will continue as heretofore. The senior editor is now in Europe, and we hoped to receive an editorial letter from him in time for this issue—a hope which tardy mails, and other causes, have disappointed. H.

